# Functional Insight of Nitric-Oxide Induced DUF Genes in *Arabidopsis thaliana*

**DOI:** 10.3389/fpls.2020.01041

**Published:** 2020-07-14

**Authors:** Rizwana Begum Syed Nabi, Rupesh Tayade, Qari Muhammad Imran, Adil Hussain, Muhammad Shahid, Byung-Wook Yun

**Affiliations:** ^1^ Laboratory of Plant Functional Genomics, School of Applied Biosciences, Kyungpook National University, Daegu, South Korea; ^2^ Department of Southern Area Crop Science, National Institute of Crop Science, Rural Development Administration, Miryang, South Korea; ^3^ Laboratory of Plant Breeding, School of Applied Biosciences, Kyungpook National University, Daegu, South Korea; ^4^ Department of Agriculture, Abdul Wali Khan University, Mardan, Pakistan

**Keywords:** Arabidopsis, transcriptome, DUF569, differentially expressed genes, nitro-oxidative stress

## Abstract

Advances in next-generation sequencing technologies facilitate the study of plant molecular functions in detail and with precision. Plant genome and proteome databases are continually being updated with large transcriptomic or genomic datasets. With the ever-increasing amount of sequencing data, several thousands of genes or proteins in public databases remain uncharacterized, and their domain functions are largely unknown. Such proteins contain domains of unknown function (DUF). In the present study, we identified 231 upregulated and 206 downregulated DUF genes from the available RNA-Seq-based transcriptome profiling datasets of *Arabidopsis* leaves exposed to a nitric oxide donor, *S*-nitroso-L-cysteine (CysNO). In addition, we performed extensive *in silico* and biological experiments to determine the potential functions of *AtDUF569* and to elucidate its role in plant growth, development, and defense. We validated the expression pattern of the most upregulated and the most downregulated DUF genes from the transcriptomic data. In addition, a loss-of *AtDUF569* function mutant was evaluated for growth, development, and defense against biotic and abiotic stresses. According to the results of the study, *AtDUF569* negatively regulates biotic stress responses and differentially regulates plant growth under nitro-oxidative stress conditions.

## Introduction

Nitric oxide (NO) is a gaseous and highly reactive free radical involved as a signaling molecule in vital physiological processes in both animal and plant cells. Over the past few decades, various functional aspects of NO in animal and plant cell biology have been explored and described. NO in plants has increasingly emerged as an essential molecule involved in diverse plant functions, such as seed germination, growth, and development, plant defense, iron homeostasis, biotic and abiotic stresses, senescence, and cell death (reviewed by Domingos et al., 2015; Nabi et al., 2019) signaling pathways of indole acetic acid, abscisic acid (ABA), and other plant hormones (García-Mata and Lamattina, 2002; Pagnussat et al., 2003). Despite the importance and involvement of NO in diverse plant functions, its origin and mechanism of production in higher plants remain largely elusive.

One of the key roles of NO in the regulation of protein function is through posttranslational modifications such as *S*-nitrosation (or *S*-nitrosylation) and tyrosine nitration (Stamler et al., 1992; Greenacre and Ischiropoulos, 2001). Chief among them is *S*-nitrosation, the covalent attachment of NO to exposed cysteine residues of proteins to form *S*-nitrosothiols (Stamler et al., 1992). Such protein modification has been reported to play critical roles in cellular processes, enzyme pathways, protein–protein interactions, and protein localization (Tada, 2008; Wang et al., 2009; Yun et al., 2011).

In the current genomic era, advances in sequencing technologies are generating vast quantities of data at the transcriptomic and genomic levels. Although the genomes of several mammals, higher plants, and microbial species have been sequenced and annotated, several genes remain uncharacterized, and their biochemical and biological functions, in turn, remain unknown. Such uncharacterized proteins are deemed as proteins having domains of unknown function (DUF) and have been categorized in the Pfam database as a protein family with no functional annotation (Bateman et al., 2010; Finn et al., 2016; El-Gebali et al., 2019). Despite extensive research, DUF is one of the largest families in the Pfam database, representing more than 22% of the entire domains in the Pfam database, with approximately 4,000 DUFs that are poorly understood (Bateman et al., 2010; Finn et al., 2016).

Studies suggest that DUF domain-containing proteins play a vital role in plant stress responses. For instance, in wheat, the DUF622 domain-containing *TaSRG* (*Triticum aestivum* salt response gene) increased salt tolerance in overexpressed transgenic *Arabidopsis* and influenced gene expression levels under salt stress in rice (He et al., 2011). Similarly, in *Arabidopsis*, ABA and drought-induced ubiquitin ligase genes, *AtRDUF1* and *AtRDUF2* (RING-DUF1117E3), regulate ABA signaling and drought stress, while single and double knockout (KO) mutants of *AtRDUF1* and *AtRDUF2* exhibited reduced tolerance to ABA-mediated drought stress when compared with the wild type (WT) plants (Kim and Kim, 2013). Similarly, in another study on rice plant, Cui et al. (2016) reported that *DUF1645* was upregulated in response to various stress factors and conferred improved drought tolerance when overexpressed.

Furthermore, negative regulation of DUF genes has also been reported. For example, in rice, the *DUF966* domain-containing *OsDSR2* encodes a protein that negatively regulates responses to salt and simulated drought stress, along with ABA signaling (Luo et al., 2014). Recently, the functions and expression levels of various members of the rice *OsDUF866* family (*OsDUF866.1*–*4*) were analyzed (Li et al., 2017). Reports suggested that *OsDUF866.1* positively regulated heat stress tolerance, while *OsDUF866.2* expression decreased discernibly following exposure to drought conditions. *OsDUF866.3* was highly expressed in response to drought and cold stress and decreased under heat stress, while *OsDUF866.4* expression was upregulated under cold and heat stress and decreased under drought stress. In addition, the roles of other DUF genes in rice, such as *OsDUF872*, *OsDUF829*, and *OsDUF946*, have been described (Li et al., 2017; Li et al., 2018a; Li et al., 2018b).

Microarray and RNA-seq mediated transcriptome analyses have made it convenient to predict the global changes in gene expression in a particular genotype. A number of transcriptome analyses in response to various stress conditions have been conducted including H_2_O_2_-mediated oxidative stress (Desikan et al., 2001), ozone (Xu et al., 2015) cold, drought and oxidative stress (Sham et al., 2014). Nitrosative stress-induced changes in transcriptional regulation have been studied extensively. Microarray or RNA-Seq profiling has revealed several key genes with differential expression in response to 1 mM *S*-nitrosoglutathione (GSNO) and 0.1 and 1.0 mM sodium nitroprusside (SNP) in *Arabidopsis* (Parani et al., 2004; Begara-Morales et al., 2014). At the same time, such transcriptome data revealed several genes with unknown functions, which have exhibited differential expression in response to nitrosative stress. In our previous study involving *Arabidopsis* leaves, 1 mM *S*-nitroso-L-cysteine (CysNO)-mediated RNA-Seq-based transcriptome analysis revealed the differential expression of several thousand of genes (Hussain et al., 2016). In the present study, we identified approximately 440 DUF genes that exhibited differential responses to CysNO, including 235 upregulated and 205 downregulated genes in *Arabidopsis thaliana*, using high-throughput RNA-Seq data. We subsequently characterized them by *in silico* analysis to decipher any putative roles of the genes. In addition, we also studied the biological role of *AtDUF569* in plant growth, development, and defense, using a functional genomics study. Our aim was to identify and characterize NO-induced genes encoding DUFs, with an emphasis on *AtDUF569*.

## Materials and Methods

### Transcriptome-Wide Identification of DUF Genes and Validation of RNA-Seq Data

In a previous study, we reported approximately 6436 differentially expressed genes (DEGs), among which 440 were identified as containing DUFs (Hussain et al., 2016). The DUF name is assigned based on the functional annotation information. When the function of the protein has not been determined, it is deposited in the Pfam database (El-Gebali et al., 2019). Such proteins/genes are commonly termed DUFs (Bateman et al., 2010; Punta et al., 2012). All the 440 DUF domain containing DEGs exhibited significant (*p* ≤ 0.05) differential expression in response to 1 mM CysNO. The list of the DUF domain-containing DEGs was examined carefully for any duplicate values. To visualize the differential expression of the DUFs between the CysNO-treated and the untreated (control) samples, a heatmap with hierarchical clustering presented as a dendrogram was generated using *R* version 3.3.1.R (https://www.r-project.org/) using the fragments per kilobase of transcript per million mapped read (FPKM) values.

To validate the RNA-Seq data of the upregulated and downregulated DEGs qRT-PCR was performed as described previously (Imran et al., 2018a). Briefly, total RNA was extracted from *A. thaliana* leaf samples 6 h after 1 mM CysNO infiltration. The cDNA was synthesized using Biofact RT kit (BioFACT™, Korea) according to the manufacturer’s standard protocol. A two-step qRT-PCR reaction was performed using 2× Real-Time PCR Master mix (including SYBR Green I; BioFACT™) in an Illumina Eco Real-Time PCR system (Illumina™, USA). The PCR conditions were initial denaturation at 95°C for 15 min, followed by 40 cycles at 95°C for 20 s and 60°C for 40 s. The fold change in transcript accumulation of the selected genes was compared with that of the RNA-Seq data. Pearson’s correlation coefficient (*R*) for both data sets was calculated using MS Excel 2016 (Microsoft Corp., Redmond, WA, USA). The primers used in the present study are listed in **Table S1**.

### Functional Annotation and Gene Ontology Analysis of DUF Genes

To determine the NO-induced DUF genes, we used the Phytozome database (https://phytozome.jgi.doe.gov/pz/portal.html) to find the corresponding pathways and biological information. The CysNO-induced DUF domain-containing DEGs were analyzed for associated Gene Ontology (GO) terms that describe biological processes, molecular functions, and cellular components through the PANTHER v14.1 (http://pantherdb.org) web interface, using the applicable search field and *A. thaliana* selected as the organism. The “statistical overrepresentation test” was selected, and the rest of the analyses were performed using default settings. Only the GO terms with significant (p < 0.05) fold enrichment were selected, and pie charts were constructed for visualization using MS Excel 2016.

### Promoter Analysis

To forecast the putative regulatory role of NO-responsive DUF genes, we obtained the promoter sequences 1 kb upstream of the transcription initiation site of the selected NO-responsive *Arabidopsis* DUF genes from The *Arabidopsis* Information Resource Center (TAIR) (https://www.arabidopsis.org/). The retrieved sequences were analyzed for potential cis-regulatory elements through the web-based MatInspector tool (https://omictools.com/matinspector-tool) with default settings. Only the transcription factor (TF) binding sites vital in plant regulatory responses to biotic and abiotic stresses were selected for visualization.

### Phylogenetic Analysis

To examine the evolutionary relationship of the NO-responsive DUF DEGs, a list of 18 DUF DEGs including 10 upregulated and 8 downregulated (two downregulated genes were transposons) was used as query in Phytozome against rice (*Oryza sativa*), soybean (*Glycine max*), wheat (*Triticum aestivum*), maize (*Zea mays*) and tomato (*Solanum lycopersicum*). The protein sequences of these orthologs were retrieved from Phytozome web interface (https://phytozome.jgi.doe.gov/phytomine/begin.do) and aligned using ClustalW in Mega 7 (Kumar et al., 2016), the resultant alignment was used to make phylogenetic tree using maximum likelihood based on JTT matrix-based model (Jones et al., 1992) with bootstrap value of 1,000 replicates. All of the positions with less than 95% site coverage were eliminated; that is, fewer than 5% alignment gaps, missing data, and ambiguous bases were allowed at any position. MEGA 7.0 (Kumar et al., 2016) was used for the analysis.

### Plant Material and Growth Conditions

The seeds of WT *A. thaliana* (ecotype Col-0) and the mutant line *atduf*569 (AT1G69890) were obtained from the Nottingham Arabidopsis Stock Centre (NASC; http://arabidopsis.info/). In addition, *atgsnor1-3* knockout mutant was used as disease susceptible control due to its established role in plant immunity (Feechan et al., 2005). Similarly, the *atsid2* mutant deficient in the SA pathway was used as a control for SA-mediated defense pathway, and *atcat2,* a high H_2_O_2_-producing mutant deficient in *AtCATALASE2* gene (AT1G74710; Hu et al., 2010) was used as susceptible controls for the nitro-oxidative stress assay and biotic stress-related experiments. Plants homozygous for T-DNA insertion were identified through genotyping using PCR with the T-DNA left border primers and gene-specific primers obtained from Signal Salk (http://signal.salk.edu/tdnaprimers.2.html; **Figure S1**, **Table S1**). The PCR conditions were an initial denaturation at 94°C for 2 min, followed by 30 cycles of 94°C for 20 s, 58°C for 30 s, 72°C for 1 min, and a final extension at 72°C for 5 min. The confirmed homozygous mutant line was used for experimental biological evaluation after collecting the seeds in bulk. All the seeds were surface-sterilized using 50% bleach (commercial bleach) with 1% (*v*/*v*) Triton X-100 added, for about 1–2 min. After rinsing five times, the sterilized seeds were incubated in sterile water and stratified overnight at 4°C for uniform germination before sowing either on half-strength Murashige and Skoog (MS) medium (Murashige and Skoog, 1962) or soil.

### Nitro-Oxidative Stress Assay

The responses of *AtDUF569* (*AT1G69890*) to nitro-oxidative stress in plants were evaluated as described by Imran et al. (2016). Briefly, for nitro-oxidative stress conditions, Col-0 (WT), *atgsnor1-3* (*AT5G43940*), *atcat2* (AT1G74710), and *atduf*569 (AT1G69890) seeds were sterilized (See section *Plant material and growth conditions*) and grown on half-strength MS medium supplemented with 0.75 mM CysNO or GSNO as NO donors and 2 mM H_2_O_2_ or 1 µM MV as oxidative stress inducers, separately. The control and the treated plants were grown under 16/8 h light/dark conditions at 23 ± 2°C. The experiments were performed in triplicate and cotyledon development frequency (CDF) was measured after seven days, as described previously (Imran et al., 2018b). In addition, root and shoot lengths were measured 14 days after sowing.

### Pathogen Inoculations and Pathogenicity Assessment

Plants were subjected to pathogen inoculation as described previously (Yun et al., 2011). Briefly, *Pseudomonas syringae* pv. *tomato* virulent (*Pst* DC3000) and avirulent strains (*Pst* DC30000 *avrB*) were used for the pathogenicity assays. Bacterial cultures were grown and maintained as described previously (Whalen et al., 1991). Briefly, a single colony was cultured in Luria–Bertani (LB medium) broth with respective antibiotics added and incubated at 28°C overnight with shaking. The bacterial cultures (1 ml each) were centrifuged at 8,000 rpm (LABOGene 1736R) for 3 min. The bacterial pellet was resuspended in 1 ml of 10 mM MgCl_2_ solution. The prepared bacterial inoculum was then infiltrated into the abaxial side of the leaves *via* a needleless syringe at 5 × 10^5^ CFU ml^−1^ for virulent *Pst* DC3000 and 0.002 at OD_600_ (1 × 10^6^ CFU ml^−1^) for avirulent *Pst* DC30000 *avrB*. Mock plants were infiltrated with the 10 mM MgCl_2_ solution without bacteria. Leaf samples were collected over time for further gene expression analyses. In addition, leaf discs from *Pst DC3000*-inoculated leaves were crushed in 10 mM MgCl_2_ and diluted 10 times. To identify the responses of *atduf569* and control genotypes to infection, the diluted samples were spread on LB agar plates containing the respective antibiotics. The development of symptoms was observed, and photos were captured using a digital camera (EOS 700D, Canon). Symptom development and pathogen growth in plant tissues were assessed 1, 2, and 3 d post-inoculation (dpi) by counting the bacterial CFU per leaf disc using the serial dilution method as described by Flors et al. (2008), and disease symptoms on the inoculated leaves were also observed.

### Electrolyte Leakage

Cell death induced by pathogen infection was assessed through electrolyte leakage (Arasimowicz-Jelonek et al., 2009). Briefly, triplicate leaf discs (about 1 cm^2^) with three leaf discs per replicate were collected from the plants inoculated with the avirulent bacterial pathogen (*Pst* DC3000 *avrB*) at an OD_600_ of 0.02 corresponding to 1 × 10^6^ CFU ml^−1^. The leaf discs were washed three times with deionized water and maintained in approximately 300 µl of deionized water/well in a six-well plate (SPL Life Sciences, Korea). Electrolyte leakage of each sample was recorded over several time points using a portable conductivity meter (Huriba Twin Cond B-173).

### Statistical Analysis

The data were analyzed at *p* ≤ 0.05 or *p* ≤ 0.01 significance levels. A two-tailed *t*-test was performed to determine *p*-values using MS Excel 2016. The means and standard errors were calculated from at least three biological replicates and compared with the control.

## Results

### Transcriptome-Wide Identification and Characterization of NO-Responsive DUF Genes

**Figure 1:**
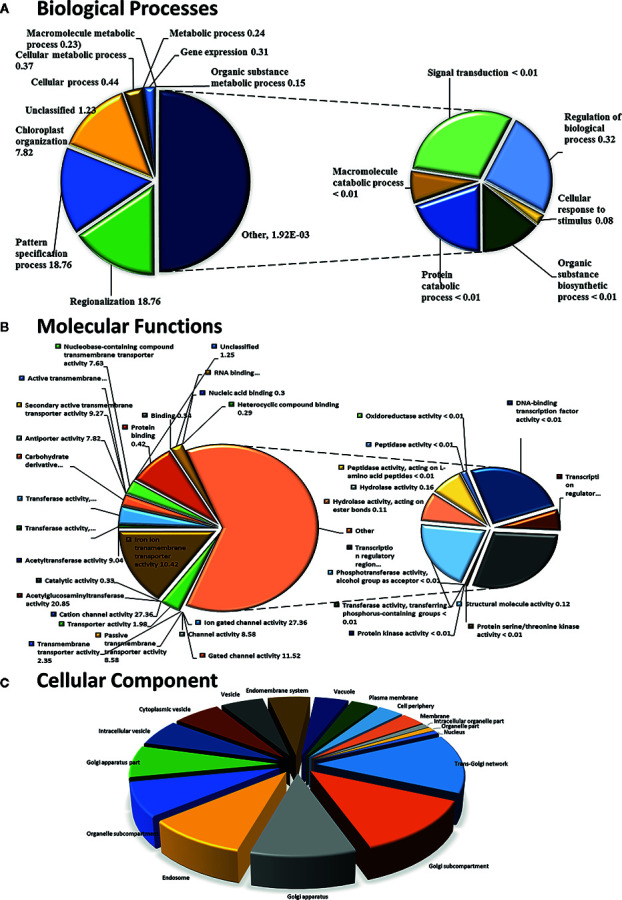
Functional GO term classification of CysNO-responsive DUF genes of *Arabidopsis* leaf transcripts. Gene ontologies for **(A)** Biological Processes **(B)** Molecular Functions and **(C)** Cellular Process were determined using *Arabidopsis thaliana* as a reference genome. Out of the 443 CySNO responsive genes, 440 were mapped with the reference genome, PANTHER version 14.1 (http://pantherdb.org) at *P* < 0.05 for the PANTHER GO-slim and the fold enrichment-values shown.

**Figure 2:**
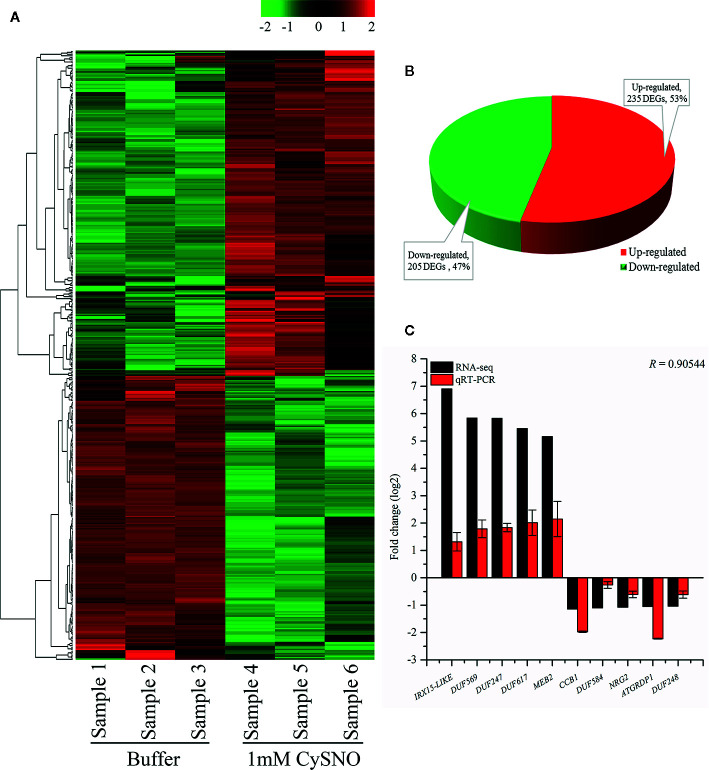
Identification and analysis of NO-responsive DUF domain-containing genes. **(A)** Heatmap revealing the expression pattern of transcriptome-wide, DEGs in response to *S*-nitroso L cysteine (CySNO) and hierarchical clustering generated from the FPKM values of CysNO-responsive DUF domain-containing genes. Red and green colors indicate the expression values in the respective sample. Samples 1to 3 are buffer-treated while 4 to 6 are CySNO infiltrated. A color key representing the intensities of the expression values is also given. **(B)** Pie-chart illustrating the total 440 number of up- and downregulated NO-responsive DUF domain-containing DEGs; these gene were identified at a Q-value < 0.05 in the transcriptome. **(C)** qRT-PCR validation of RNA-Seq data. About 10 DUF domain-containing genes (five each from up- and downregulated DEGs) were selected based on their fold change for validation through qRT-PCR. The *R* value represents Pearson’s correlation coefficient and was calculated among RNA-Seq and qRT-PCR datasets. The data points in C are the mean of three replicates while error bars represent standard error.

To identify NO-responsive DUF domain-containing genes, we analyzed CysNO-induced DEGs reported previously (Hussain et al., 2016). All the RNA-seq data can be found in the public repository for Gene Expression Omnibus (GEO) and Short Read Achieve (SRA) under accession numbers GSE81361 and SRP074890, respectively. Notably, a considerable number (437 DEGs) of NO-responsive DUF domain-containing genes were differentially expressed in response to 1 mM CysNO (**Supplementary Table S2**). A list of top 10 up- and down-regulated DEGs is given in **Table 1**. We analyzed all the NO-responsive DUFs for associating Gene Ontology (GO) terms. GO terms for biological process suggested that NO responsive DUFs were associated with chloroplast organization, pattern specification process, cellular metabolic process and gene expression *etc*. (**Figure 1A**). In GO terms for molecular functions majority of DUFs were associated with Iron ion transmembrane transporter activity (**Figure 1B**). In GO terms of cellular component majority of DUFs were associated with Golgi apparatus, Golgi subcomponent, and Endosomes *etc.* (**Figure 1C**). A heatmap with hierarchical clustering between the control and the CysNO-treated samples was constructed from the FPKM values in triplicates and shows the expression patterns of the DUF domain-containing DEGs (**Figure 2A**). Samples 1 to 3 represent three replicates from control (buffer only) while samples 4 to 6 represent three samples from CySNO treated plants. The red color in the treated samples indicates upregulated genes, while the intensity of the color represents the intensity of expression. The more the red color, the more expression after CySNO treatment (**Figure 2A**). Some samples with black color show very low expression approaching to zero (**Figure 2A**). Among the 437 DUF domain-containing DEGs, 53% (236 DEGs) were upregulated, while 47% (205 DEGs) were downregulated, with at least a two-fold change in their expression relative to the control plants (**Figure 2B**, **Supplementary Table S2**). Furthermore, we evaluated the role of the NO-induced *DUF569* (AT1G69890) in plant growth and defense against biotic and abiotic stresses. *AtDUF569* had a fold change of 57.29 in the transcriptome profile.

**Table 1:** List of top 10 up- and downregulated NO-responsive DUF domain containing genes with their fold change as per transcriptomic analysis.

Accession No.	FPKM(1)	FPKM(2)	Fold change	p-value	Log2 (Fold change)	Annotation
AT4G10290	0.02000	27.65000	1382.50000	0.00006	10.70240	RmlC-like cupins superfamily protein; CONTAINS InterPro DOMAIN/s: Protein of unknown function DUF861, cupin-3 (InterPro : IPR008579), Cupin, RmlC-type (InterPro : IPR011051), RmlC-like jelly roll fold (InterPro : IPR014710); BEST *Arabidopsis thaliana* protein match is: RmlC-like cupins superfamily protein (TAIR : AT4G10280.1)
AT3G43250	0.11000	60.46000	549.63636	0.00000	9.12997	Family of unknown function (DUF572); CONTAINS InterPro DOMAIN/s: Protein of unknown function DUF572 (InterPro : IPR007590); BEST *Arabidopsis thaliana* protein match is: Family of unknown function (DUF572) (TAIR : AT2G32050.1)
AT1G62320	0.03000	3.99000	133.00000	0.00000	7.24010	ERD (early-responsive to dehydration stress) family protein; FUNCTIONS IN: molecular_function unknown; INVOLVED IN: biological_process unknown; LOCATED IN: endomembrane system, membrane; EXPRESSED IN: pollen tube; CONTAINS InterPro DOMAIN/s: Protein of unknown function DUF221 (InterPro : IPR003864); BEST *Arabidopsis thaliana* protein match is: ERD (early-responsive to dehydration stress) family protein (TAIR : AT1G11960.1)
AT5G67210	0.82000	98.38000	119.97561	0.00000	6.90488	Encode a DUF579 (domain of unknown function 579) containing protein essential for normal xylan synthesis and deposition in the secondary cell wall.
AT5G41590	0.04000	4.76000	119.00000	0.00000	6.87891	Protein of unknown function (DUF567); CONTAINS InterPro DOMAIN/s: Protein of unknown function DUF567 (InterPro : IPR007612); BEST *Arabidopsis thaliana* protein match is: Protein of unknown function (DUF567) (TAIR : AT2G38640.1)
AT2G14290	0.28000	23.59000	84.25000	0.00000	6.37693	CONTAINS InterPro DOMAIN/s: F-box domain, cyclin-like (InterPro : IPR001810), F-box domain, Skp2-like (InterPro : IPR022364), Protein of unknown function DUF295 (InterPro : IPR005174); BEST *Arabidopsis thaliana* protein match is: F-box family protein with a domain of unknown function (DUF295) (TAIR : AT5G25290.1); Has 351 Blast hits to 340 proteins in 13 species: Archae—0; Bacteria—2; Metazoa—0; Fungi—0; Plants—349; Viruses—0; Other Eukaryotes—0 (source: NCBI BLink).
AT1G54095	0.02000	1.63000	81.50000	0.00842	6.04292	Protein of unknown function (DUF1677); CONTAINS InterPro DOMAIN/s: Protein of unknown function DUF1677, plant (InterPro : IPR012876); BEST *Arabidopsis thaliana* protein match is: Protein of unknown function (DUF1677) (TAIR : AT1G72510.2)
AT4G36820	0.12000	7.70000	64.16667	0.00000	6.01626	Protein of unknown function (DUF607); CONTAINS InterPro DOMAIN/s: Protein of unknown function DUF607 (InterPro : IPR006769); BEST *Arabidopsis thaliana* protein match is: Protein of unknown function (DUF607) (TAIR : AT2G23790.1); Has 370 Blast hits to 370 proteins in 122 species: Archae—0; Bacteria—0; Metazoa—148; Fungi—54; Plants—129; Viruses—0; Other Eukaryotes—39 (source: NCBI BLink).
AT3G04300	0.08000	5.03000	62.87500	0.00000	5.96407	RmlC-like cupins superfamily protein; CONTAINS InterPro DOMAIN/s: Cupin, RmlC-type (InterPro : IPR011051), Protein of unknown function DUF861, cupin-3 (InterPro : IPR008579), RmlC-like jelly roll fold (InterPro : IPR014710); BEST *Arabidopsis thaliana* protein match is: RmlC-like cupins superfamily protein (TAIR : AT4G10300.1); Has 512 Blast hits to 512 proteins in 136 species: Archae—0; Bacteria—273; Metazoa—0; Fungi—0; Plants—140; Viruses—0; Other Eukaryotes—99 (source: NCBI BLink).
AT1G69890	1.92000	110.000	57.29167	0.00000	5.83694	CONTAINS InterPro DOMAIN/s: Protein of unknown function DUF569 (InterPro : IPR007679), Actin cross-linking (InterPro : IPR008999); BEST *Arabidopsis thaliana* protein match is: Actin cross-linking protein (TAIR : AT1G27100.1)
AT4G14819	2.77000	0.03000	92.33333	0.00943	−6.71390	Protein of unknown function (DUF1677); CONTAINS InterPro DOMAIN/s: Protein of unknown function DUF1677, plant (InterPro : IPR012876); BEST *Arabidopsis thaliana* protein match is: Protein of unknown function (DUF1677) (TAIR : AT3G22540.1); Has 35333 Blast hits to 34131 proteins in 2444 species: Archae—798; Bacteria—22,429; Metazoa—974; Fungi—991; Plants—531; Viruses—0; Other Eukaryotes—9,610 (source: NCBI BLink).
AT5G05840	0.48000	0.01000	48.00000	0.00258	−5.43983	Protein of unknown function (DUF620); CONTAINS InterPro DOMAIN/s: Protein of unknown function DUF620 (InterPro : IPR006873); BEST *Arabidopsis thaliana* protein match is: Protein of unknown function (DUF620) (TAIR : AT3G55720.1); Has 1807 Blast hits to 1807 proteins in 277 species: Archae—0; Bacteria—0; Metazoa—736; Fungi—347; Plants—385; Viruses—0; Other Eukaryotes—339 (source: NCBI BLink).
AT5G32621	0.42000	0.02000	21.00000	0.00140	−4.10636	transposable element gene; similar to unknown protein [*Arabidopsis thaliana*] (TAIR : AT3G15310.1); similar to hypothetical protein 24.t00017 [*Brassica oleracea*] (GB : ABD64939.1); contains InterPro domain Protein of unknown function DUF635; (InterPro : IPR006912); contains InterPro domain Bacterial adhesion; (InterPro : IPR008966)
AT3G54260	2.43000	0.12000	20.25000	0.00000	−4.37071	Encodes a member of the TBL (TRICHOME BIREFRINGENCE-LIKE) gene family containing a plant-specific DUF231 (domain of unknown function) domain. TBL gene family has 46 members, two of which (TBR/AT5G06700 and TBL3/AT5G01360) have been shown to be involved in the synthesis and deposition of secondary wall cellulose, presumably by influencing the esterification state of pectic polymers. A nomenclature for this gene family has been proposed (Volker Bischoff & Wolf Scheible, 2010, personal communication).
AT5G25460	95.13000	6.13000	15.51876	0.00000	−3.95598	Protein of unknown function, DUF642; FUNCTIONS IN: molecular_function unknown; INVOLVED IN: response to karrikin; LOCATED IN: plant-type cell wall; EXPRESSED IN: 22 plant structures; EXPRESSED DURING: 13 growth stages; CONTAINS InterPro DOMAIN/s: Protein of unknown function DUF642 (InterPro : IPR006946); BEST Arabidopsis thaliana protein match is: Protein of unknown function, DUF642 (TAIR : AT5G11420.1); Has 1,807 Blast hits to 1,807 proteins in 277 species: Archae—0; Bacteria—0; Metazoa—736; Fungi—347; Plants—385; Viruses—0; Other Eukaryotes—339 (source: NCBI BLink).
AT1G11700	39.37000	2.64000	14.91288	0.00000	−3.89880	Protein of unknown function, DUF584; FUNCTIONS IN: molecular_function unknown; INVOLVED IN: biological_process unknown; LOCATED IN: chloroplast; EXPRESSED IN: 21 plant structures; EXPRESSED DURING: 11 growth stages; CONTAINS InterPro DOMAIN/s: Protein of unknown function DUF584 (InterPro : IPR007608); BEST *Arabidopsis thaliana* protein match is: Protein of unknown function, DUF584 (TAIR : AT1G61930.1); Has 334 Blast hits to 333 proteins in 24 species: Archae—0; Bacteria—0; Metazoa—0; Fungi—4; Plants—328; Viruses—0; Other Eukaryotes—2 (source: NCBI BLink).
AT2G34170	10.84000	0.74000	14.64865	0.00000	−3.86850	Protein of unknown function (DUF688); FUNCTIONS IN: molecular_function unknown; INVOLVED IN: biological_process unknown; CONTAINS InterPro DOMAIN/s: Protein of unknown function DUF688 (InterPro : IPR007789); BEST *Arabidopsis thaliana* protein match is: Protein of unknown function (DUF688) (TAIR : AT1G29240.1)
AT4G12980	107.85	7.84000	13.75638	0.00000	−3.78144	Auxin-responsive family protein; CONTAINS InterPro DOMAIN/s: Uncharacterized conserved protein UCP037471 (InterPro : IPR017214), Cytochrome b561, eukaryote (InterPro : IPR004877), Protein of unknown function DUF568, DOMON-like (InterPro : IPR007613), DOMON related (InterPro : IPR005018), Cytochrome b561/ferric reductase transmembrane (InterPro : IPR006593); BEST *Arabidopsis thaliana* protein match is: Auxin-responsive family protein (TAIR : AT3G25290.2); Has 675 Blast hits to 673 proteins in 107 species: Archae—0; Bacteria—4; Metazoa—91; Fungi—93; Plants—473; Viruses—0; Other Eukaryotes—14 (source: NCBI BLink).
AT1G03300	2.99000	0.24000	12.45833	0.00000	−3.62457	Member of the plant-specific DUF724 protein family. Arabidopsis has 10 DUF724 proteins. Loss of function mutant has a WT phenotype
AT3G15310	25.97000	2.10000	12.36667	0.00000	−3.62950	transposable element gene; similar to unknown protein [*Arabidopsis thaliana*] (TAIR : AT5G32621.1); similar to hypothetical protein 24.t00017 [*Brassica oleracea*] (GB : ABD64939.1); contains InterPro domain Protein of unknown function DUF635; (InterPro : IPR006912); contains InterPro domain Bacterial adhesion; (InterPro : IPR008966)

### Validation of RNA-Seq Data Through qRT-PCR

The transcriptional changes in the expression of DUF domain-containing genes were validated by analyzing the 10 representative DEGs (five upregulated and five downregulated) through qRT-PCR. The genes were selected based on the highest fold change from both upregulated and downregulated genes in response to CysNO infiltration. The expression patterns in qRT-PCR corroborated the RNA-Seq results for all of the 10 analyzed *DUF* domain-containing DEGs. The RNA-Seq and qRT-PCR datasets were compared based on the Pearson’s correlation coefficient (*r* = 0.905), revealing a high similarity between the datasets (**Figure 2C**).

### Promoter Analysis for the Identification of the TF Binding Sites

We analyzed the promoter region of selected NO-induced DUF genes 1 Kb upstream of the transcription initiation site and the *cis*-elements or TF binding sites that were involved in plant stress were subsequently mapped (**Figure S2**). A detailed list of these TF binding sites is given in **Supplementary Table S3**. We identified the critical binding sites, including sites for the prolamin box (P-box), which was observed in 100% of the analyzed sequences. Similarly, two other elements, the W-box (WRKY TF family) and MAD-box (RIN), were found in 100% of the analyzed sequences. The AP2L ethylene-responsive TF was found in 54.1%, ETTIN (Auxin Response Factor 3) in 70.8%, ethylene-responsive TF ERF017 in 83.3%, ATMYB77-binding site in 83.3%, GT-box elements (GT2) in 70.8%, ANT (*Arabidopsis* protein AINTEGUMENTA) in 29.1%, SP8BF in 62.5%, HSFA1A in 95.8%, CDM1 in 29.6%, TATA-box in 91.6%, ANAC087 in 95.8%, and HBP1B in 91.6% of the analyzed sequences, respectively (**Figure S2**; **Table S3**). Among the above major elements, AP2L recognizes motifs within pathogenesis-related promoters, which may mediate the regulation of gene expression under biotic stress and components of stress signal transduction pathways (Xie et al., 2019). Similarly, other elements, such as ATMYB77 (an R2R3-type MYB TF), WRKY20, and ANAC087 (a NAC domain-containing protein), have been reported to play important roles in biotic and abiotic stresses. Therefore, the identification of regulatory elements and their modules is a prerequisite step for the understanding of gene expression and regulation (Nuruzzaman et al., 2013).

### Phylogenetic Analysis of *Arabidopsis At*DUF569 (AT1G69890)

**Figure 3:**
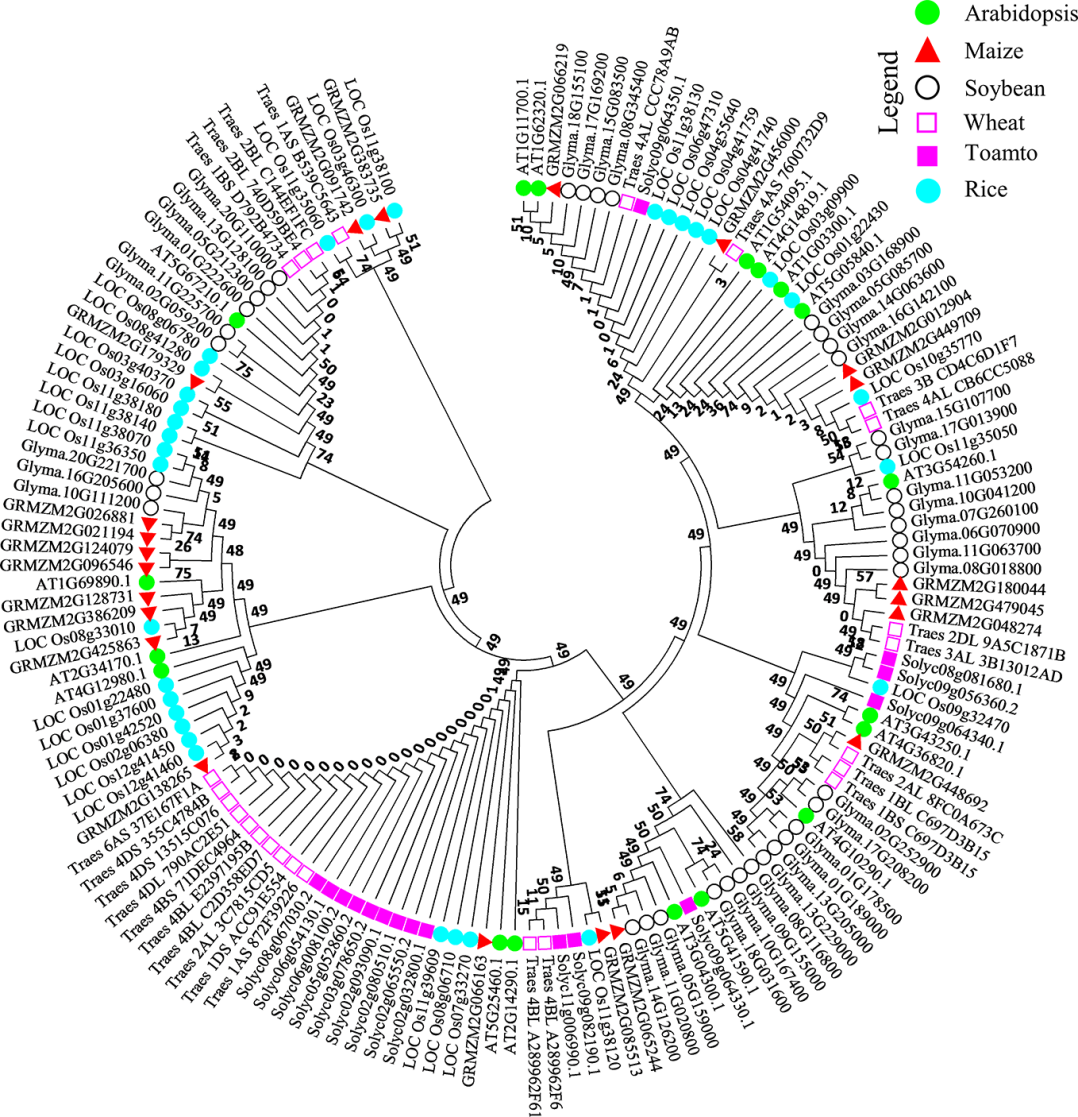
Molecular phylogenetic analysis of the AtDUF569 protein with homologs from other plant species. About 18 NO-responsive DUF DEGs were used as query against rice, soybean, wheat, maize, and tomato databases in Phytozome. The best-hit orthologs were identified and sequences were retrieved and aligned using ClustalW in Mega 7. The resultant alignment was used to make phylogenetic tree using maximum likelihood method on JTT matrix-based model through which evolutionary history was inferred. The bootstrap value was 1,000 replicates and branches corresponding to partitions reproduced in less than 50% of bootstrap replicates were eliminated. About 151 peptide sequences from Arabidopsis and various other crops mentioned above were used for this analysis. The positions with less than 95% site coverage were collapsed. The phylogenetic tree was constructed using Mega 7 software. Different species are labeled with different shapes and colors and can be found in the legend in the up-right corner of the figure.

To observe the ancestral evolutionary relationship of Arabidopsis DUF genes Top 10 upregulated and downregulated DEGs were used as query to identify their orthologs in agronomically important species such as rice, soybean, wheat, maize, and tomato. About 151 protein sequences from all the species were used to generate a phylogenic tree. From the analysis, we can predict that Arabidopsis DUFs are distributed almost in every clade. All the peptides can be divided into four major clades and subclades. From an evolutionary perspective, rice and Arabidopsis were distributed throughout the tree, interestingly, majority of the wheat proteins were grouped together with tomato and were evolutionarily distant from Arabidopsis, rice, and soybean (**Figure 3**) Together these results suggest that these species may share common ancestors and that NO-responsive DUF protein functions could be conserved in these species.

### Interactome of CysNO-Induced DUF Gene (AT1G69890)

**Figure 4:**
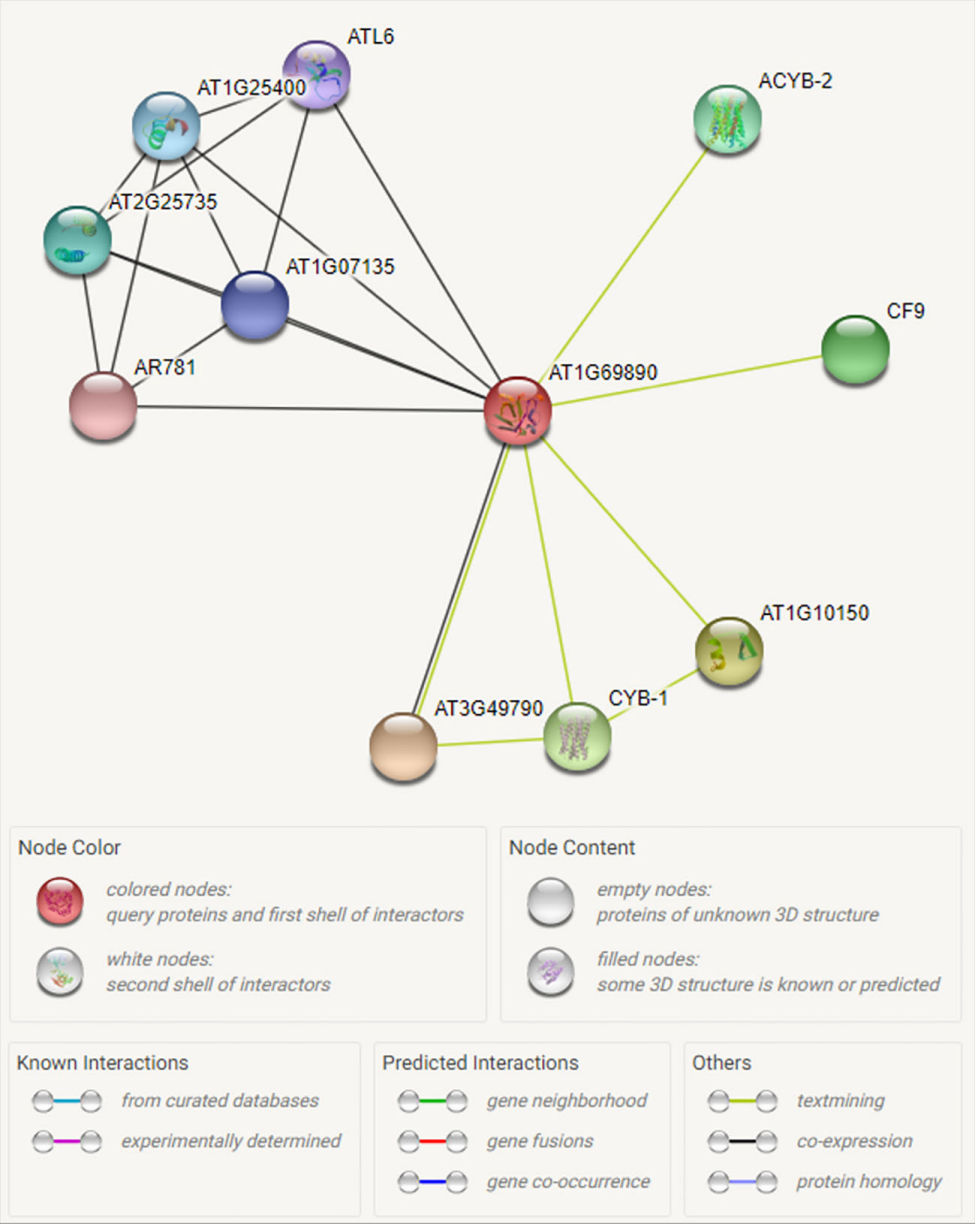
Interactome of the CysNO-induced AtDUF569. To predict possible interaction of ATDUF569 with other proteins, the accession number of DUF569 was used as “Protein name” in the search option in online Search Tool for the Retrieval of Interacting Genes/Proteins (STRING; https://string-db.org/) with default parameters. A list of interacting proteins with DUF569 is given in **Table S2** with relevant information. Different colors represent proteins or various types of interactions.

We have searched for interactions between the CysNO-induced *AtDUF569* (AT1G69890) and other proteins using the Search Tool for the Retrieval of Interacting Genes/Proteins (STRING; https://string-db.org/). We observed some interesting interactions between the DUF569 and other proteins, and we identified 10 predicted functional partners, which included the uncharacterized protein, AT3G49790, known as a carbohydrate-binding protein. Its function is described as ATP-binding, but the underlying biological mechanism remains unknown. The PHLOEM PROTEIN 2-LIKE A10 is another carbohydrate-binding protein located in the mitochondria and found in several plant species and at different growth stages. The CYB-1 and ACYB-2 proteins are potentially transmembrane ascorbate ferrireductase 2, which contains two-heme-cytochrome (1 and 2) and is involved in the catalyzation of ascorbate-dependent transmembrane ferric-chelate reduction. Such interactions of DUF569 protein with other important proteins are illustrated in **Figure 4** and **Table S4**.

The *in silico* and experimental observations suggested that the AtDUF569 protein interacts with various proteins involved in cellular trafficking machinery and carbohydrate-binding and with glycine-rich proteins that participate in cellular stress responses and signaling (Czolpinska and Rurek, 2018).

### *AtDUF569* Differentially Regulates Plant Shoot Growth and Negatively Regulates Root Growth Under Nitro-Oxidative Stress Conditions

**Figure 5:**
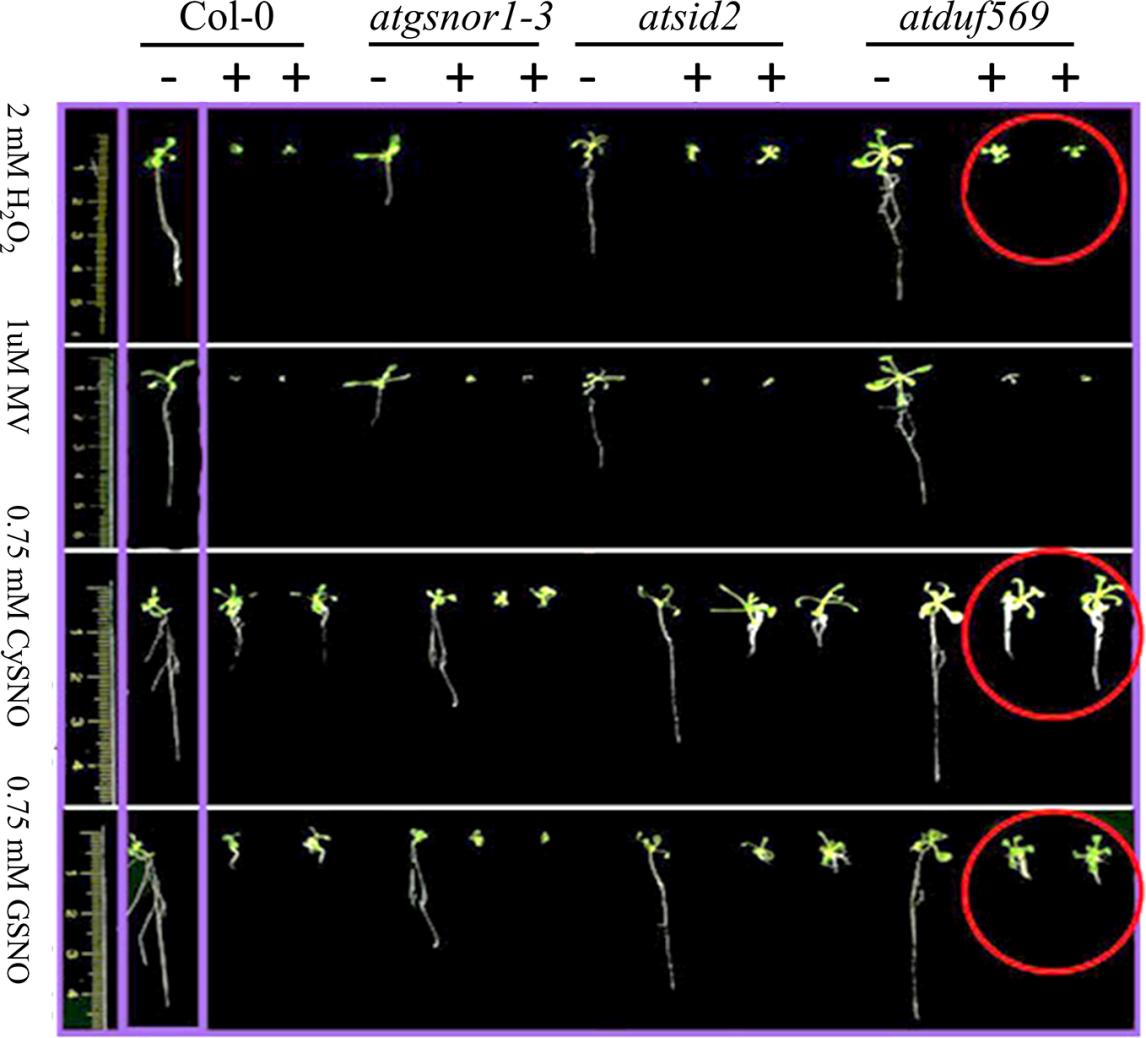
Differential role of *AtDUF569* in plant development. Phenotypic evaluation of the genotypes under the control (−) and following exposure to various oxidative and nitrosative stresses (+). For the control genotype, a single plant is presented, whereas two plants are illustrated for the treatment.

**Figure 6:**
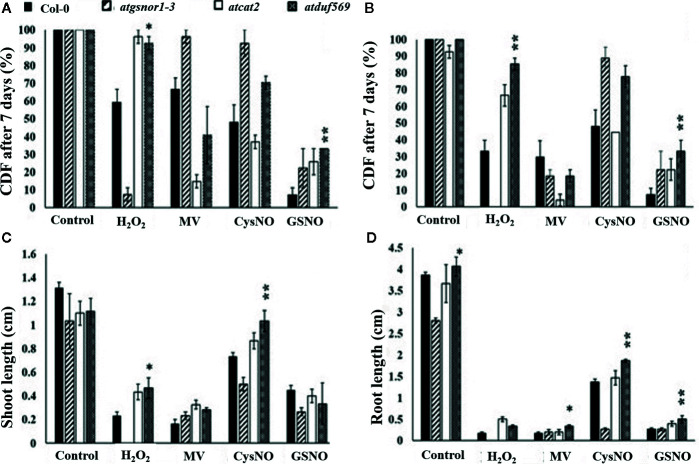
Exogenous application of CysNO, GSNO, or H_2_O_2_ and MV mediated nitro-oxidative stress. Designated genotypes Col*-*0, *atgsnor1–3*, *atcat2*, and *atdfu569*, were grown on half-strength Murashige and Skoog (MS) medium, with and without supplementation with either H_2_O_2_ and methyl viologen (Medina-Rivera et al., 2015) or CysNO and *S*-nitrosoglutathione (GSNO) for oxidative and nitrosative stress conditions, respectively. **(A)** Cotyledon development frequency (CDF) of the designated genotypes seven days after sowing under indicated stress. **(B)** CDF of the designated genotypes 14 days after sowing under indicated stress. **(C)** Shoot lengths of the designated genotypes 14 days after indicated stress. **(D)** Root lengths of the designated genotypes 14 days after indicated stress. All data points represent the means of triplicates. The experiment was repeated three times, with similar results. Error bars represent the standard error. Significant interactions are indicated by an asterisk (Student’s *t*-test with a 95% confidence level).

We explored and applied the CDF as a development or fitness score for each plant. In the data recorded after seven days of treatment, our KO mutant *atduf569* exhibited a noticeably higher CDF compared with Col-0 during exposure to CysNO- and GSNO-induced nitrosative stress, while all plants shared similar CDF result 14 days post-treatment. However, regarding MV-induced oxidative stress, KO mutant exhibited markedly reduced CDF at both seven- and 14-days post-stress treatment. On the contrary, under H_2_O_2_ exposure, the CDF was markedly higher for the KO mutant than for the Col-0 plants at both seven and 14 days after stress treatment (**Figures 5** and **6**). The results suggested that the *atduf569* mutant was tolerant to CysNO- and GSNO-mediated nitrosative stress but was considerably tolerant and sensitive to H_2_O_2_ and MV, respectively. We also measured other plant attributes, such as shoot and root length, of the mutant in comparison to the WT (Col-0) and other designated control mutants. The KO mutant *atduf569* exhibited higher shoot length under oxidative stress (induced by H_2_O_2_ and MV) 14 days post stress treatment relative to the other plants. However, shoot and root length displayed differential patterns under different nitrosative stress conditions. In addition, shoot and root lengths of KO mutant *atduf569* were higher under CysNO, while the KO mutant under GSNO stress presented reduced length and increased shoot and root lengths, respectively (**Figures 5** and **6**). Overall, the results revealed that *AtDUF659* (AT1G69890) differentially regulates plant growth and development under nitro-oxidative stress conditions.

### *AtDUF569* Negatively Regulates Plant Basal Defense at Early Time Point

**Figure 7:**
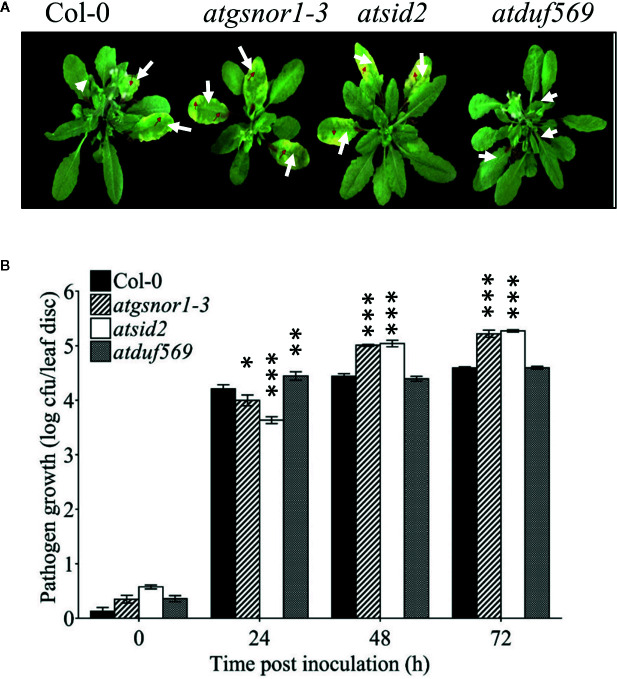
*AtDUF569* negatively regulates basal defense. **(A)** Development of symptoms post-inoculation with virulent *Pst* DC3000, **(B)** Growth of the virulent *Pst* DC3000 in plants.

**Figure 8:**
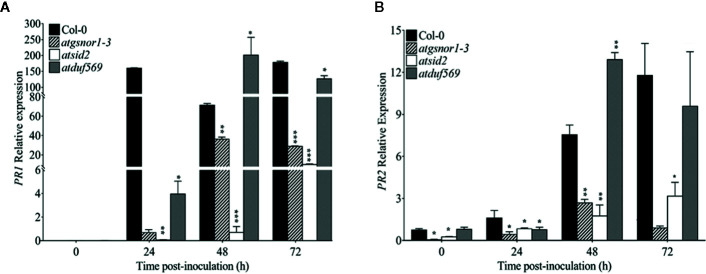
Relative expression levels of *PR1* and *PR2* in basal defense. **(A)** Relative expression of *PR1* gene after *Pst* DC3000 (virulent) inoculation, **(B)** Relative expression of *PR2* gene after *Pst* DC3000 (virulent) inoculation. The experiments were conducted with three replicates and repeated four times, with similar results. Data were analyzed using the Student’s *t*-test: *p ≤ 0.05, **p ≤ 0.01, ***p ≤ 0.001.

To examine the role of *AtDUF569* (AT1G69890) in plant basal defense, we observed the phenotypic response of the *atduf569* KO mutant line under the control conditions, as well as post pathogen inoculation as described elsewhere (Koornneef et al., 1991). The *atduf569* KO mutant line was in the Col-0 genetic background; therefore, WT Col-0 plants were used as a control, while *atgsnor1–3* KO mutant was used as a disease-susceptible control (Feechan et al., 2005). The loss-of-function mutant for *Salicylic acid induction-deficient 2* (*SID2*) inhibits pathogen-induced salicylic acid (SA) production and makes the plants highly susceptible to infection by pathogens. Therefore, *atsid2* was used as a second control for pathogenicity assessment. The pathogenicity assay revealed that the *atduf569* KO line exhibited disease symptoms at 1 dpi, but in the later assessment, it was completely resistant toward the *Pst* DC3000 infection and exhibited no disease symptoms compared with Col-0 (WT), *atgsnor1–3*, and *atsid2* plants (**Figure 7A**). Similar results were observed for the bacterial CFU counts at 3 dpi (**Figure 7B**). Based on the results, we hypothesized that the enhanced resistance to pathogens in the *atduf569* line could have been due to the upregulation of the SA signaling pathway. Therefore, we performed an expression analysis for *Pathogenesis-related 1* (*PR1*) and *PR2* genes, which are regulators of SA-dependent gene expression. The qRT-PCR analysis revealed that the transcript accumulations of *PR1* and *PR2* were significantly (*P* < 0.05) lower in *atduf569* than in the WT (Col-0) but higher than those of recognized sensitive genotypes, *atgsnor1–3* and *atsid2*, 24 *h* post-inoculation (hpi) and 48 hpi, at which it displayed the highest expression. At 72 dpi, however, the expression level was lower in comparison to the expression level in WT, although the level was higher than in recognized sensitive genotypes *atgsnor1–3* and *atsid2* (**Figures 8A, B**).

### *AtDUF569* Study of Effector-Triggered Immunity or *R-*Gene Mediated Resistance

**Figure 9:**
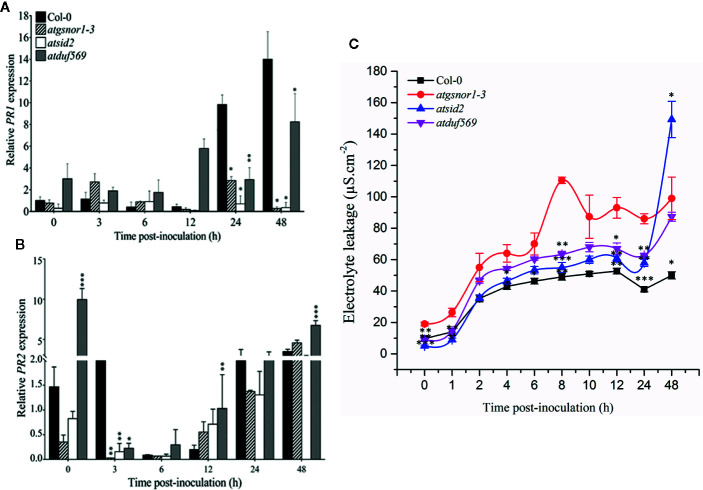
Relative expression of *PR1* and *PR2* during evaluation of the genotypes for R-gene-mediated resistance. Plants were inoculated with the *Pst DC3000 avrB*, and symptoms were recorded for analyzing the gene expression of the pathogenesis-related *PR*s genes. **(A)** *PR1*, **(B)** *PR2* expression, **(C)** Electrolyte leakage over time. All the data points are the mean of three replicates. Data were analyzed using the Student’s *t*-test: *p ≤ 0.05, **p ≤ 0.01, ***p ≤ 0.001.

To examine whether the *AtDUF569* function is required for *R*-gene mediated resistance, we inoculated plants with *Pst* DC3000 *avrB*. Our results suggested early, high transcript accumulation of the in *atduf569* when compared to the WT plants (**Figure 9A**) at 0, 3, 6, and 12 hpi. On the contrary, *PR2* expression was significantly (*P* < 0.05) lower relative to the WT at 3 hpi with a gradual increase in expression at 6, 12, 24, and 48 hpi (**Figure 9B**). The observation indicated that *PR2* transcript accumulation might have a higher contribution to the control of pathogen growth at later time points. Furthermore, the electrolyte leakage assay, performed after *Pst* DC3000 *avrB* inoculation, revealed higher electrolyte leakage in the *atduf569* plants over time relative to the other genotypes except for *atgsnor1-3* (**Figure 9C**).

### *AtDUF569* Study for Systemic Acquired Resistance-Mediated Resistance

**Figure 10:**
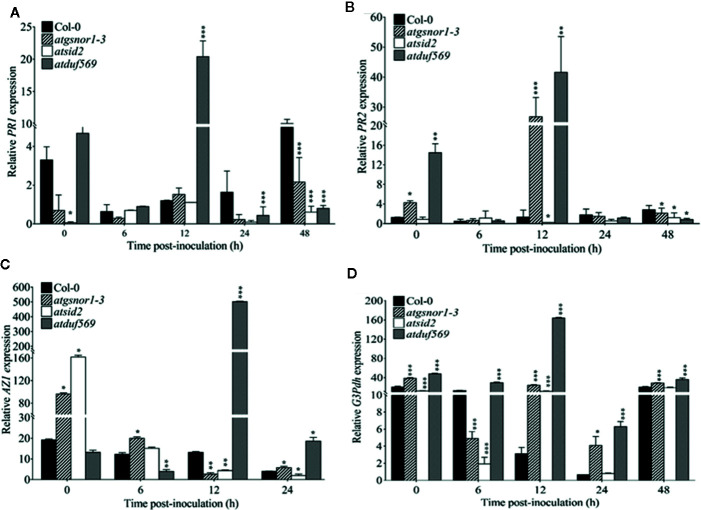
Relative expression of *PR1*, *PR2*, *AZI*, and *G3DPH*. A*tDUF569* negatively regulates systemic acquired resistance. Plants were inoculated with the *Pst DC3000 avrB*, and symptoms were recorded to analyze the gene expression dynamics of the pathogenesis-related genes. All the data points are the mean of three replicates **(A)** *PR1*, **(B)** *PR2*, **(C)** *AZI*, and **(D)** *G3DPH*. Data were analyzed using the Student’s *t*-test: *p ≤ 0.05, **p ≤ 0.01, ***p ≤ 0.001.

In plants, signaling molecules, such as azelaic acid, glycerol-3-phosphate, and SA-*PR1* and -*PR2*, are involved in mediating SAR in plants. The expression patterns of *PR1* and *PR2* revealed a significantly (*P* < 0.05) higher level of transcript accumulation in a*tduf569* at 0, 6, and 12 hpi but a drastically decreased level at 24 and 48 hpi in comparison to WT, *atgsnor1–3*, and *atsid2* plants (**Figures 10A, B**). In addition, the expression levels of *AZI* were significantly (*P* ≤ 0.05) higher at 12 and 24 hpi and *G3pdh* at 0, 6, 12, 14, and 48 hpi (**Figures 10C, D**). Based on the expression pattern, the initial upregulation of *PR1* and *PR2* genes at 0, 6, and 12 hpi and the differential upregulation of *AZI* and *G3pdh* potentially contributed to the *atduf569* mutant plants’ resistance to the *Pst* DC3000 infection. The results provide further evidence that the *atduf569*-resistant phenotype responded *via* the upregulation of SA-dependent *PR*s at the initial stage of pathogenicity, which controlled the effects of the pathogen and protected the mutant phenotype from later-stage disease development.

## Discussion

NO is a small, diatomic, and highly reactive gaseous molecule that orchestrates a plethora of physiological functions both in plants and animals. Although, its role has been well-explored in the mammalian system (Kerwin et al., 1995), NO opened a new pandora-box in plant science when it was reported as a signaling molecule during plant defense (Delledonne et al., 1998; Durner et al., 1998). In plants, contrary to other signaling cascades, NO transfer its bio-activity *via* posttranslational modifications (PTMs) such as *S*-nitrosation (Stamler et al., 2001), tyrosine nitration (Ischiropoulos et al., 1992), and metal nitrosylation (Gupta et al., 2011). These PTMs are known to regulate protein function under various stress conditions; however, it used to be very difficult to predict that a particular protein may undergo NO-mediated PTMs. The evolution of *in silico* tools such as GPS-SNO (Xue et al., 2010) made it quite easy to predict if a protein may undergo S-nitrosation or not. Similar tools are available for other PTMs too. Global gene expression such as RNA-seq has made it easy to identify candidate genes that are responsive to a stimulus. In the last couple of decades, a number of transcriptomes have revealed a bulk of data that generated interesting information about NO-mediated transcriptional changes. For example, transcriptome analysis of Arabidopsis leaves and roots in response to 1 mM GSNO exogenous application differentially expressed about 3,263 genes after 3 h of treatment (Begara-Morales et al., 2014). Similarly, Parani et al. (2004) showed 422 DEGs in response to 0.1 mM sodium nitroprusside (SNP). Recently in RNA-seq mediated transcriptome analysis we reported more than six thousand genes (Hussain et al., 2016) and transcription factors (Imran et al., 2018a) that showed differential expression in response to 1 mM CySNO. In the present study, using a transcriptome-wide search, we identified a total of 440 NO-responsive DUF genes that showed differential expression in response to 1 mM CysNO. Among the 437 DUF domain-containing DEGs, 231 (53%) were upregulated, while 206 (47%) were downregulated, with at least a two-fold change in their expression, implying that the genes have a key role in transcriptional regulation of various physiological processes. The differential expression of such huge number of DUF genes suggests their involvement in NO biology and possibly in NO-mediated post-translational modifications. *In silico* analyses of DUF569 for targeting potential sites for phosphorylation suggest at least five different sites in DUF569 that could undergo phosphorylation (**Figure S3B**). Similarly, the 3D protein structure and simulation by GPS SNO for identification of potential sites for S-nitrosylation also suggested the presence of exposed Cysteine residues that could be potential target for S-nitrosylation (**Figures S3B, C**). DUF genes have also been identified in response to other stimuli. In Arabidopsis transcriptomic profiling in response to 0.1 and 1.0 mM SNP, 126 genes (98 upregulated and 28 downregulated) with unknown functions were reported (Parani et al., 2004). Similarly, a transcriptomic study of *Arabidopsis* root exposed to 250 µM SNP revealed the largest category of genes with unknown and unclassified proteins known to date (Badri et al., 2008). More recently, in other plant species, such as upland cotton, transcriptome analysis based on Taq sequencing found 265 genes with uncharacterized proteins (Huang et al., 2018). Individual DUF genes have also been characterized. In a transcriptomic study of *Arabidopsis* plants exposed to SNP and O_3_, there was a more than two-fold change in expression and the upregulation of *AtDUF569* (AT1G69890) (Ahlfors et al., 2009), suggesting that the *AtDUF569* participates in O_3_‐induced cell death and NO production.

We observed a significant increase in the expression of *AtDUF569* (57-fold) in response to NO donor application, which indicates relatively upstream and direct involvement of *AtDUF569* in NO-related responses in *A. thaliana*. The presence of the P-box binding site for prolamin box-binding factor 1 (pbf1) and W-box sequences in the promoters of all the DUF genes was particularly interesting because W-box sequences have been recognized as positive regulators of senescence in the Minghui 63 rice variety, in which they are bound by leaf senescence-specific proteins (Liu et al., 2016). Furthermore, the W-box is the cognate *cis*-element for WRKY proteins, which regulates essential cellular functions (Imran et al., 2018b). The presence of other *cis*-elements, such as RIN, the GT-box, ANT, HSF, and TATA, also indicates the involvement of DUF genes in key physiological processes. TFs are involved in plant development and defense, inducing or repressing the transcription of specific genes in signaling pathways that ultimately control the differential responses toward plant growth and defense (Jin et al., 2017). Reports have suggested that NO-responsive genes contain a significantly higher number of certain transcription factor binding sites (TFBS) in their promoter regions (Palmieri et al., 2008). Palmieri et al. (2008) using in silico approach analyzed 28447 Arabidopsis genes and suggested that several TFBS were found at least 15% more often in NO-induced genes. Furthermore, promoter analysis of other non-NO-responsive DUF genes may help scientists understand the full potential of the gene family. The functions of DUF genes in plants may well be highly similar and evolutionarily conserved, as indicated by the high homology observed during phylogenetic analysis of DUF genes from different plant species, which could be one of the reasons why the exact functions of the genes remain elusive, as one would expect different functions in different plant species; simple/model or complex.

Through the predicted interactome analysis, we observed that *AtDUF569* could interact with potentially important proteins. For example, out of ten proteins, DUF569 interacted with AT3G49790 which helps in ATP binding, therefore AtDUF569 is suggested to have important role during carbohydrate metabolism. Similarly, another interacting protein ATL6 E3 ubiquitin protein ligase is reported to be involved in regulating early steps of plant defense signaling, suggesting that AtDUF569 may regulate plant defense functions directly or by interacting with other proteins. Another interesting interacting protein is AR781 which is a pheromone receptor-like protein (DUF1645); if characterized fully, this gene might be a key in entomological research to attract a particular type of insects such as the honeybee. Thus, the interaction of AtDUF569 yields interesting and important information that can be further explored by an in-depth study.

We further sought to determine the role of DUF genes in the biological system using functional genomics approach. We, therefore, selected DUF569 which was among the top 10 DUFs that had the highest fold change in response to CysNO. Our results suggested that DUF569 negatively regulates shoot and root growth under nitro-oxidative stress, while in the case of pathogenicity, *duf569* showed sensitive phenotype at an early time point (24 h) followed by more pathogen growth while it showed a resistant phenotype afterward (**Figures 7A, B**). This was in accordance with the PR gene expression which showed low expression at 24 h while significantly higher expression at 48 h.

The trend indicates that the *atduf569*-resistant phenotype could be due to the upregulation of SA-dependent *PR* genes at the initial stage of pathogenicity, which influences the effect of the pathogen effect and, in turn, protects the mutant phenotype from later-stage toxicity and disease symptoms.

Collectively, our results suggested the possible involvement of DUF genes in plant metabolism and energy generation and/or turnover involving active trafficking between cellular organelles. Furthermore, biological data on *atduf569* KO plants suggested the involvement of the gene in regulating plant shoot and root growth besides plant responses to oxidative as well as nitro-oxidative stress conditions.

## Conclusion and Future Prospects

The current study illustrates the role of *AtDUF569* in plant biology using a functional genomics approach. To the best of our knowledge, this is the first study attempting to functionally characterize NO-induced DUF genes in *Arabidopsis.* To date, no study has attempted to characterize NO-induced DUF genes in plant systems. Our study presents an exemplary model for future transcriptomic studies on similar DUF genes or proteins to elucidate molecular and functional aspects in detail in different plant species. This study motivates further studies to aid the comprehensive characterization of *AtDUF569* about its cellular functioning.

## Data Availability Statement

The datasets generated for this study can be found in the the public repository for Gene Expression Omnibus (GEO) and Short Read Achieve (SRA) under accession numbers GSE81361 and SRP074890.

## Author Contributions

RN performed the experiments and analyzed the data. RN, RT, and QI drafted the manuscript. AH and MS helped with the conceptualization of the study. QI, MS, and RN prepared illustrations, figures, tables, and references. B-WY, AH, and QI edited the manuscript. B-WY contributed critical comments to the draft and approved the manuscript. All authors contributed to the article and approved the submitted version

## Funding

This work was supported by Next-Generation Bio Green21 Program (SSAC, Grant No. PJ01342501) Rural Development Administration, South Korea.

## Conflict of Interest

The authors declare that the research was conducted in the absence of any commercial or financial relationships that could be construed as a potential conflict of interest.
