# Supplementary material for: Functional Insight of Nitric-Oxide Induced DUF Genes in Arabidopsis thaliana
Source: Front Plant Sci. 2020 Jul 14;11:1041. doi: 10.3389/fpls.2020.01041 (PMC7378322; doi:10.3389/fpls.2020.01041)
Supplement: Supplementary file 1 [file DataSheet_1.zip › Supplementary material/Supplementary material.DOCX]

**Supplementary Material**

**Functional insight of nitric-oxide induced DUF569 gene in Arabidopsis thaliana**

Rizwana Begum Syed Nabi^#1^, Rupesh Tayade^#2^, Qari Muhammad Imran^#1^, Adil Hussain^3^, Muhammad Shahid^1^, , Byung-Wook Yun^1^*

* Correspondence: Corresponding Author: [bwyun@knu.ac.kr](mailto:bwyun@knu.ac.kr)


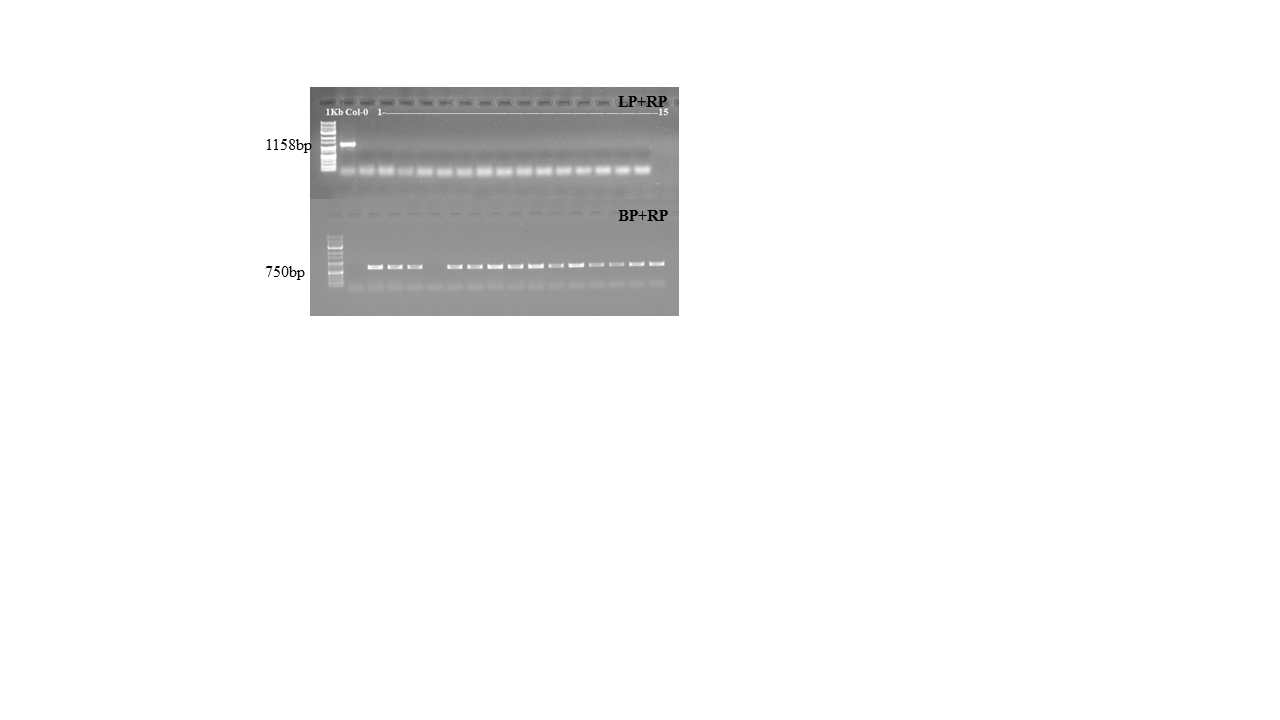


**Figure S1 The identification of homozygous lines through PCR Col-0: wild type**; **1-15 *atduf569* mutant plants with *atduf569(-/-)* allele.**


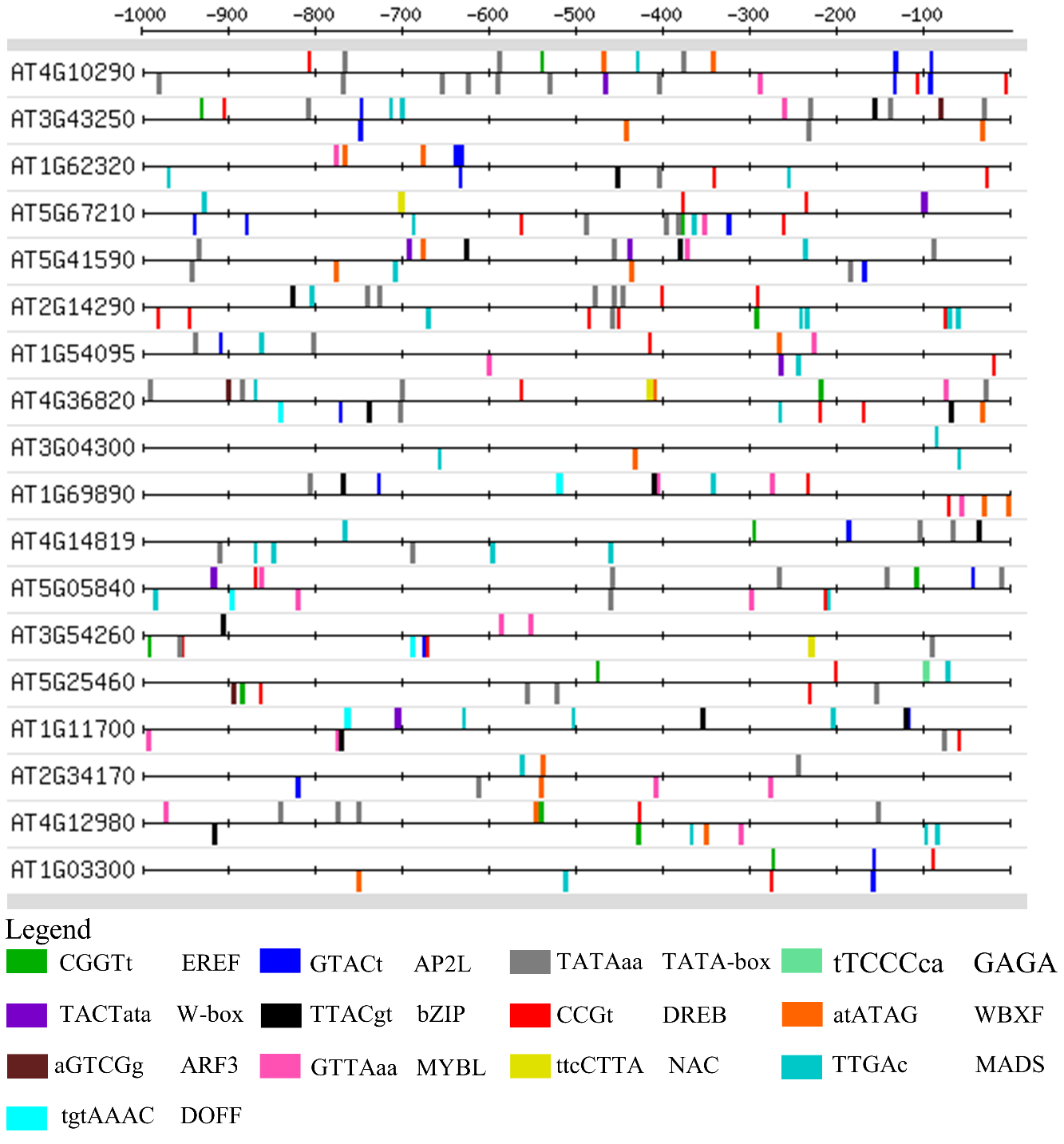


**Figure S2 Promoter analysis for predicting *cis* regulatory elements**

A total of 20 (10 each up- and down-regulated) NO-responsive DUF domain-containing genes were selected for promoter analysis. Promoter sequences 1 Kb upstream of the transcription initiation site were retrieved from The Arabidopsis Information Resource (TAIR) and were analyzed using the MatInspector online web interface (<https://omictools.com/matinspector-tool>) for prediction of cis-regulatory elements and mapped through the regulatory sequence analysis tool. Two of the down-regulated genes (AT3G15310 and AT5G32621) were transposons and therefore their sequences could not be retrieved hence were not included in the analysis.


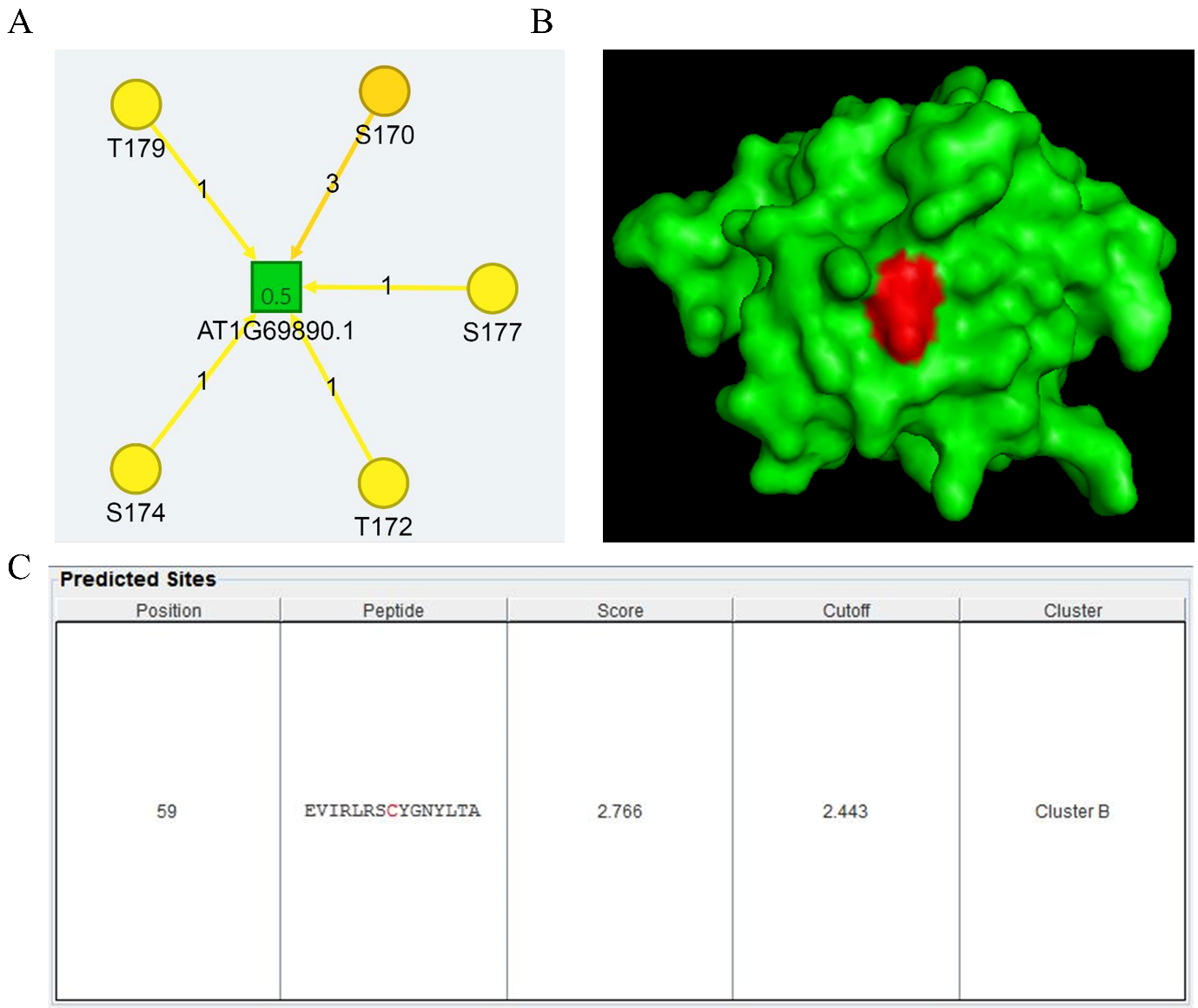


**Figure S3 proteomic properties of DUF569**

(A). Potential phosphorylation sites in DUF569 (B) 3D protein structure of DUF569, with exposed Cystein residue (red) which could be target for protein S-nitrosylation (C) prediction of S-nitrosylation site in DUF569 using high treshhold level in GPS-SNO software

**Table S1 Primers for qRT-PCR to validate RNA-Seq data and relative expression analysis**

| S.No | Accession No. | Gene Name | Forward sequence | Reverse sequence |
| --- | --- | --- | --- | --- |
| 1 | AT3G18780 | *Actin* | GCTGGACGTGACCTTACTGA | CCATCTCCTGCTCGTAGTCA |
| 2 | AT5G67210 | *DUF579* | ACCACCGACTCCATCATCTC | AGCATTGCATTGATGGCTGT |
| 3 | AT1G69890 | *DUF569* | TTGTCTAAACCGGCTCGACT | TTGGCTCGAAGCAAGTTTCC |
| 4 | AT5G22540 | *DUF247* | ACGTAGCTTTCATGGCTTGT | CGGGACACTTCATCCTCTGT |
| 5 | AT5G06990 | *DUF617* | GAACCGATTTGGGCGTTGTA | CATCGACACAGCATGAAGCA |
| 6 | AT5G24290 | *DUF125* | TTCAACCGGACGGAGAAAGA | AACCAGAGGCAGAAGCAGAT |
| 7 | AT3G26710 | *CCB1* | AGCAAGTGTTGGTCTTGTCT | CCTCTTTCCTCGACGCCTTT |
| 8 | AT3G45210 | *DUF584* | GCTCCGATTCGCCTGAATTT | CCAGTTCTGTACGTTCATCGG |
| 9 | AT3G60320 | *DUF630* | GTACGGCGTTGTAAGGATCG | GGCGAATGAGGAAAGAGCAG |
| 10 | AT2G22660 | *DUF1399* | CGAGTCGCTGTCTCGTTTAC | AGAAGCAAGAGCCTTTGCAC |
| 11 | AT1G19430 | *DUF248* | GCTTATTTCGCGCGTAGGAT | AGACGAAGCAGAGACCCAAA |
| 12 | AT2G14610 | *PR1* | GTGCAATGGAGTTTGTGGTC | TCACATAATTCCCACGAGGA |
| 13 | AT3G57260 | *PR2* | CAGATTCCGGTACATCAACG | AGTGGTGGTGTCAGTGGCTA |
| 14 | AT2G41540 | *AtG3Pdh* | AAATATGTCGAGGCAAGGCT | CCACACAGCTTCTTGCAGAT |
| 15 | AT4G12470 | *AZI* | GCAAGCCAAGTCCTAAACCA | GTCGACGTCAACCAAACCTT |
| 16 | AT1G69890 | *DUF569* | TCGACGTCTTTTTATTTGAACG | TACGCATCCTTACGGCTTTAG |

**Table S4 Interactome of CysNO-induced DUF gene (AT1G69890) using STRING, a database of known and predicted protein–protein interactions**

| **S.N** | **Predicted functional partners** | **Description** |
| --- | --- | --- |
| 1 | AT3G49790 | Carbohydrate-binding protein. Its function is described as ATP-binding, but its role in biological processes is not yet known |
| 2 | AT1G10150 | Protein PHLOEM PROTEIN 2-LIKE A10, carbohydrate-binding protein, located in mitochondrion and expressed during different growth stages. |
| 3 | CYB-1 | Probable transmembrane ascorbate ferrireductase 2, with a two-heme-cytochrome, catalyzes ascorbate-dependent transmembrane ferric-chelate reduction |
| 4 | CF9 | Carbohydrate-binding protein, encodes CF9 |
| 5 | ACYB-2 | Cytochrome b561/ferric reductase transmembrane protein family, with a two-heme-cytochrome, catalyzes ascorbate-dependent transmembrane ferric-chelate reduction. Able to use dihydrolipoic acid as an alternative substrate to ascorbate |
| 6 | AT2G25735 | Unknown protein |
| 7 | AT1G25400 | Uncharacterized protein |
| 8 | AT1G07135 | Predicted GPI-anchored protein, glycine-rich protein |
| 9 | ATL6 | E3 ubiquitin-protein ligase ATL6 may be involved in the plant C/N response and the early steps of the plant defense signaling pathway; it belongs to the RING-type zinc finger family, ATL subfamily |
| 10 | AR781 | AR781, pheromone receptor-like protein (DUF1645), unknown function |

Note- CysNO - S-nitroso-L-cysteine, DUF- Domain of unknow function
